# Selective Allocation of LC-PUFA-Containing Lipids During Vitellogenesis in Female Sichuan Taimen (*Hucho bleekeri*): Implications for Female Broodstock Rearing During Artificial Propagation

**DOI:** 10.3390/biology15131059

**Published:** 2026-07-02

**Authors:** Qinyao Wei, Yeyu Chen, Fubin Wang, Wei Shao, Yongshen Ru, Huanchao Yang, Jun Du, Zhaobin Song, Zhenming Lai, Hua Li

**Affiliations:** 1 Fisheries Research Institute, Sichuan Academy of Agricultural Sciences, Chengdu 611730, China; 2 Key Laboratory of Bio-Resources and Eco-Environment of the Ministry of Education, College of Life Sciences, Sichuan University, Chengdu 610065, China; 3 Sichuan Zumuzu River Hydropower Development Company, Ltd., Chengdu 610041, China; 4 Power China Hydropower Development Group Co., Ltd., Chengdu 610041, China; 5 Fishes Conservation and Utilization in the Upper Reaches of the Yangtze River Key Laboratory of Sichuan Province, Chengdu 611730, China

**Keywords:** lipidomics, ovarian yolk deposition, DHA, ARA, *Hucho bleekeri*

## Abstract

Oocyte quality determines fertilization success during the reproductive period of fish and is affected by a key intrinsic factor: the level of lipid deposition in the yolk. In this study, lipidomics was used to characterize lipid profiles of adipose tissue, liver, serum and ovary to clarify divergent hepatic lipid allocation to fat depots versus ovary in Sichuan taimen (*Hucho bleekeri*). The results showed that glycerophospholipids (GPs) constituted the most diverse lipid class across all four tissues, with phosphatidylcholine (PC) being the most abundant in each tissue. Relative to adipose tissue, liver, and serum, the ovary exhibited significant enrichment of PC and phosphatidylethanolamine (PE) and higher accumulation of docosahexaenoic acid (DHA)- and arachidonic acid (ARA)-containing GPs and triacylglycerols (TGs), as well as enrichment in TGs, diacylglycerols (DGs), and cardiolipin (CL), compared to adipose tissue. The expression levels of *fads6*, *elovl2*, *elovl5*, *dgat1b*, *dgat2*, *pparα*, *nfyb*, and *fabp7* were higher in the liver, while *lpcat1* was highly expressed specifically in the ovary. The results of this study demonstrate that long-chain polyunsaturated fatty acids (LC-PUFAs) exhibited selective enrichment in the ovary, which facilitates yolk lipid deposition and provides structural and energetic support for oocyte development and early embryonic development. From a lipidomic perspective, this study reveals the regulatory characteristics underlying ovarian yolk deposition in *H. bleekeri*, providing important evidence for understanding the mechanisms of egg quality formation as well as a theoretical basis for broodstock management, artificial propagation, and the improvement of egg and larval quality.

## Simple Summary

Sichuan taimen (*Hucho bleekeri*) is an endangered fish species plagued by inadequate oocyte yolk deposition during artificial reproduction. Oocyte quality largely determines fertilization efficiency in fish, and yolk lipid accumulation is a vital intrinsic factor affecting egg quality. The liver is the primary site of in vivo lipid synthesis, yet the regulatory mechanism governing hepatic lipid partitioning between adipose tissue and ovary remains unclear. In this study, lipidomics was applied to characterize lipid profiles of adipose tissue, liver, serum, and ovary. Glycerophospholipids dominated the lipid composition of all tested tissues, and the ovary was markedly enriched in phospholipids, docosahexaenoic acid (DHA)- and arachidonic acid (ARA)-containing glycerophospholipids, and triacylglycerols compared to other tissues. Key genes related to lipid synthesis and transport displayed tissue-specific differential expression between the liver and ovary. Our findings verify that long-chain polyunsaturated fatty acids are selectively accumulated in the ovary to facilitate yolk formation and sustain oocyte and early embryonic development, clarifying the underlying pattern of ovarian yolk deposition in Sichuan taimen at the lipid metabolism level and providing theoretical references for refined broodstock rearing and improved artificial propagation.

## 1. Introduction

Sichuan taimen (*Hucho bleekeri*), a member of the family Salmonidae, is an endemic and rare fish species restricted to the upper reaches of the Yangtze River in China and represents an important fishery resource. However, its populations have experienced a continuous decline in recent decades as a result of overfishing, dam construction and other reasons, which has led to its designation as a first-class national protected animal in China (National Forestry and Grassland Administration and Ministry of Agriculture and Rural Affairs of P. R. China, announcement No. 3, 2021) and as critically endangered on the International Union for Conservation of Nature (IUCN) Red List of Threatened Species [1]. To strengthen the conservation of these populations, researchers have conducted a series of studies on the species’ basic biology. For example, a female genome of *H. bleekeri* has been constructed [2], providing an important foundation for subsequent fundamental research; in immunology, genes of the IRF and MHC families have been cloned and characterized [3,4], and single-cell transcriptomic sequencing of gill tissues has been performed in response to heat stress [5]; sex-linked molecular markers have been screened and validated for sex identification [6]; and, in microbial ecology, differences in microbial communities of aquaculture pond water under different farming conditions have been compared and analyzed [7]. However, studies on lipid metabolism and lipidomics in *H. bleekeri* remain limited, and this research area is still largely unexplored.

Despite the establishment of artificial propagation techniques for *H. bleekeri* [8], the large-scale propagation of this species, similarly to many cultured fish species, is still constrained by the insufficient supply of high-quality mature eggs. Broodstocks are the foundation of successful artificial propagation; however, several prominent problems remain during the artificial breeding of *H. bleekeri*. Among these, unstable egg quality and insufficient yolk deposition, which result in low fertilization rates, poor hatching rates, low larval survival, and high deformity rates, have become major bottlenecks restricting large-scale seed production. Artificial propagation and stock enhancement are important approaches for the conservation and recovery of endangered fish resources, and unstable egg quality not only severely reduces breeding efficiency but also limits the effectiveness of wild population restoration and stock rebuilding programs. The quality of yolk deposition is closely associated with the nutritional status of broodstock, to which lipid nutrition is particularly critical, as lipids not only serve as essential energy sources for yolk formation and embryonic development but also participate in cell membrane construction, signal transduction, and organ development during embryogenesis. Therefore, elucidating the patterns of yolk lipid deposition and their nutritional regulatory mechanisms in *H. bleekeri* is of great significance for optimizing broodstock nutritional strategies, improving egg and larval quality, overcoming key bottlenecks in artificial propagation, and promoting the conservation and recovery of wild populations.

Previous studies have demonstrated that the efficiency of yolk lipid deposition is one of the key factors influencing egg quality [9]. The onset of ovarian maturation is generally considered to coincide with the completion of yolk deposition, which provides the essential energy reserves for subsequent fertilization and embryonic development. The egg of teleost fish possesses considerable yolk stores, supplying nutrient-rich proteins and lipids required for embryogenesis and subsequent larval development [10]. Numerous lipid classes, including triacylglycerols (TGs) [11,12], glycerophospholipids (GPs) [13,14], cholesterol esters (ChEs) [15,16], and mono- and diacylglycerols (MGs and DGs) [17], are closely associated with ovarian yolk deposition. Among yolk lipids, long-chain polyunsaturated fatty acids (LC-PUFAs), particularly docosahexaenoic acid (DHA, C22:6n-3) and arachidonic acid (ARA, C20:4n-6), are considered essential for oocyte maturation, membrane biogenesis, neural development, and embryonic survival in teleosts [12,18].

The ovarian yolk is presumed to be derived from the liver. In fish, the liver functions as the central organ for nutrient storage and metabolism, regulating the synthesis, mobilization, and transport of lipids and other energy substrates [19]. During ovarian maturation, it continuously supplies free fatty acids, triglycerides, and glycogen-derived metabolites to the ovaries via the bloodstream [20]; once delivered, these nutrients are transported and metabolized within ovarian cells, supporting oocyte maturation and the growth of ovarian tissues. Lipids produced in the liver can be transported through the bloodstream and stored in the form of triglycerides in adipose tissues [21], which simultaneously function as an important lipid storage depot that can mobilize endogenous fatty acids to support reproductive development [22]. Together, these tissues constitute a coordinated liver–blood–adipose/ovary axis involved in lipid allocation and yolk formation.

During vitellogenesis, lipid nutrients synthesized in the liver may be either stored in adipose tissue or selectively transported to the ovary to support yolk formation. However, the specific lipid species preferentially allocated to reproductive tissues and the molecular mechanisms underlying this tissue-specific redistribution remain poorly understood. This study aimed to explore the differences in lipid composition among the liver, adipose tissue, serum and ovary of female *H. bleekeri*, as well as the lipids specifically deposited in the ovary. The expression levels of genes related to lipid transport and synthesis were further verified by qPCR at the molecular level to clarify the characteristics of lipid transport. This work reveals the metabolic strategy of lipid allocation in female *H. bleekeri*, providing a theoretical basis for optimizing broodstock diet and improving ovarian development and reproductive performance in artificial propagation programs. Meanwhile, the findings offer important implications for the aquaculture industry, particularly for the artificial breeding and germplasm conservation of high-value cold-water fish species, and may help enhance broodstock reproductive efficiency and seed quality and facilitate the large-scale propagation and sustainable utilization of rare and endangered fish species, thereby contributing to the high-quality development of aquaculture and the sustainable exploitation of germplasm resources.

## 2. Materials and Methods

### 2.1. Sample Collection

All non-reproductive female *H. bleekeri* utilized in this experiment were artificially bred and sourced from the Fisheries Research Institute of the Sichuan Academy of Agricultural Sciences. A total of six female individuals were collected on 30 June 2025 at the Jiguanshan base in Sichuan Province. After anesthesia with MS-222, blood samples were collected from the caudal vein, and the fish were subsequently dissected to collect adipose tissue, liver, and ovary. Blood samples were allowed to clot at room temperature and then centrifuged to obtain serum, which was stored for subsequent use. During the rearing period, the fish were fed live prey every 2–3 days, mainly consisting of *Siniperca chuatsi* and *Carassius auratus*. The mean body length and weight of the sampled fish were 45.37 ± 2.18 cm and 1.25 ± 0.25 kg, respectively.

### 2.2. Lipid Extraction

For lipid extraction, 100 mg of adipose tissue, liver, serum, and ovary were placed into a glass centrifuge tube with a polytetrafluoroethylene-lined cap. After the addition of 0.75 mL pre-chilled methanol, the mixture was vortexed, 2.5 mL pre-chilled methyl tert-butyl ether (MTBE) and then 10 μL of SPLASH^TM^ internal standard (Avanti Polar Lipids, Alabaster, AL, USA) were added, and the samples were incubated at room temperature for 1 h on a shaker. Phase separation was induced by adding 0.625 mL Liquid Chromatography–Mass Spectrometry (LC-MS)-grade water, mixing, and incubating at room temperature for 10 min, followed by centrifugation at 1000 g for 10 min. The upper organic phase (MTBE) was collected, and the lower aqueous/methanol phase was re-extracted with 1 mL MTBE/methanol/water (10:3:2.5, *v*/*v*/*v*); the two organic phases were then combined and evaporated to dryness under a nitrogen stream. The dried extracts were reconstituted in 100 μL isopropanol [23] and subjected to LC-MS/MS analysis, and equal aliquots of the supernatant from each prepared sample were combined to obtain a quality control (QC) sample.

### 2.3. LC-MS/MS Analysis

Lipidomic analysis was performed using a Vanquish™ UHPLC system coupled to a Q Exactive™ HF/HF-X Orbitrap mass spectrometer (Thermo Fisher Scientific, Bremen, Germany). Lipids were separated on a Thermo Accucore C30 column (150 × 2.1 mm, 2.6 μm) maintained at 40 °C. The mobile phase consisted of solvents A (acetonitrile/water, 60:40, *v*/*v*, containing 0.1% formic acid and 10 mM ammonium acetate) and B (isopropanol/acetonitrile, 90:10, *v*/*v*, containing 0.1% formic acid and 10 mM ammonium acetate). The flow rate was 0.35 mL min^−1^, and the injection volume was 5 μL. The gradient elution program was as follows: 70% A/30% B at 0–2 min, 57% A/43% B at 5 min, 45% A/55% B at 5.1 min, 30% A/70% B at 11 min, 1% A/99% B at 16–18 min, and then initial conditions (70% A/30% B) at 18.1–20 min. Mass spectrometric data were acquired in both positive and negative electrospray ionization (ESI) modes. The sheath gas pressure was set at 40 psi; the auxiliary gas flow rate was 10 L min^−1^ and 7 L min^−1^ in positive and negative mode, respectively; the sweep gas flow rate was 0 L min^−1^; the spray voltage was 3.5 kV; the capillary temperature was 320 °C; the heater temperature was 350 °C; and the S-Lens RF level was 50. Full-scan MS spectra were acquired over an m/z range of 114–1700 with an automatic gain control (AGC) target of 3 × 10^6^ ions, and MS/MS spectra were acquired using normalized collision energies of 22, 24, and 28 eV; an isolation window of 1 *m/z*, an AGC target of 2 × 10^5^ ions; a maximum injection time of 100 ms; and a dynamic exclusion duration of 6 s.

### 2.4. Lipid Identification and Quantification

Raw data files were imported into LipidSearch for peak extraction and identification with a precursor and fragment ion mass tolerance of 5 ppm, converted to mzXML format using msconvert, and processed with XCMS for quantification, and a retention time tolerance of 0.05 min and a signal-to-noise ratio of 3 were applied. Background ions were removed using blanks, and the quantitative data were normalized. The concentration of lipid *i* was determined according to the following equation:

$$C_{ki}=C_{ls}\times \frac{Q_{ki}}{Q_{ks}}$$

In this equation, *C_ki_* denotes the concentration of lipid *i* in the sample, *C_ls_* represents the concentration of the corresponding internal standard spiked into the sample, and *Q_ki_* and *Q_ks_* correspond to the quantitative responses of lipid *i* and its associated internal standard, respectively.

### 2.5. RNA Extraction and qPCR Analysis

The liver, adipose and ovarian tissues were thoroughly homogenized in Trizol reagent using a low-temperature tissue grinder (Jingxin, Shanghai, China), and 200 μL of chloroform was subsequently added into the EP tube, followed by vigorous shaking for 30 s until the mixture became turbid. The samples were then incubated at room temperature for 2 min and centrifuged for phase separation; next, 400 μL of the supernatant was transferred to a new tube and mixed with 600 μL of pre-cooled isopropanol, and the mixture was gently inverted for 30 s, incubated for 10 min, and centrifuged again. The supernatant was discarded, and the RNA pellet was washed with 1 mL of pre-cooled 75% ethanol by gently flicking the tube, followed by centrifugation. After completely removing the residual liquid, the RNA pellet was dissolved in 10 μL of RNase-free water. The concentration and purity of total RNA were assessed using a NanoDrop spectrophotometer (Implen, Munich, Germany), and first-strand cDNA was synthesized using a reverse transcription kit (Takara, Dalian, China) according to the manufacturer’s instructions. First-strand cDNA was synthesized from 1 μL of total RNA using a reverse transcription kit (Takara, Dalian, China) according to the manufacturer’s instructions. Primer sequences are listed in Supplementary Table S1. Based on previous studies from our research group, *ef1α* was selected as the reference gene [24]. For the qPCR analysis, each biological replicate was measured in triplicate, which resulted in a total of 18 qPCR measurements per tissue (6 biological replicates × 3 technical replicates).

### 2.6. Statistical Analysis

Multivariate statistical analyses were performed using the metaX package (version 1.4.16), and principal component analysis (PCA) was conducted to calculate the variable importance in projection (VIP) values of each metabolite. For univariate analysis, statistical significances between two groups were evaluated using the Student’s *t*-test, and fold change (FC) values were calculated accordingly. A metabolite was considered significantly differential when it satisfied all thresholds: VIP > 1, *p* < 0.05, and FC ≥ 2 (up-regulated) or FC ≤ 0.5 (down-regulated).

Mfuzz clustering analysis was applied to identify variations in lipid abundance patterns across different tissues, and volcano plots were generated using the ggplot2 package in R (version 4.3.1) to integrate VIP, log_2_(FC), and −log_10_(*p*) values. Pearson correlation coefficients of differential metabolites were calculated using the cor() function in R, and the corresponding significance tests were conducted via the cor.mtest() function in the corrplot package (*p* < 0.05). Correlation networks were visualized using the same package. Differential metabolite enrichment was conducted based on the Kyoto Encyclopedia of Genes and Genomes (KEGG) and gene set enrichment analysis (GSEA).

Statistical differences among tissues were analyzed using one-way ANOVA followed by Bonferroni-corrected pairwise comparisons, and statistical significance was defined as follows: * *p* < 0.05; ** *p* < 0.01; *** *p* < 0.001; **** *p* < 0.0001; ^ns^ *p* ≥ 0.05.

## 3. Results

### 3.1. Overview of Lipid Profile

**Figure 1:**
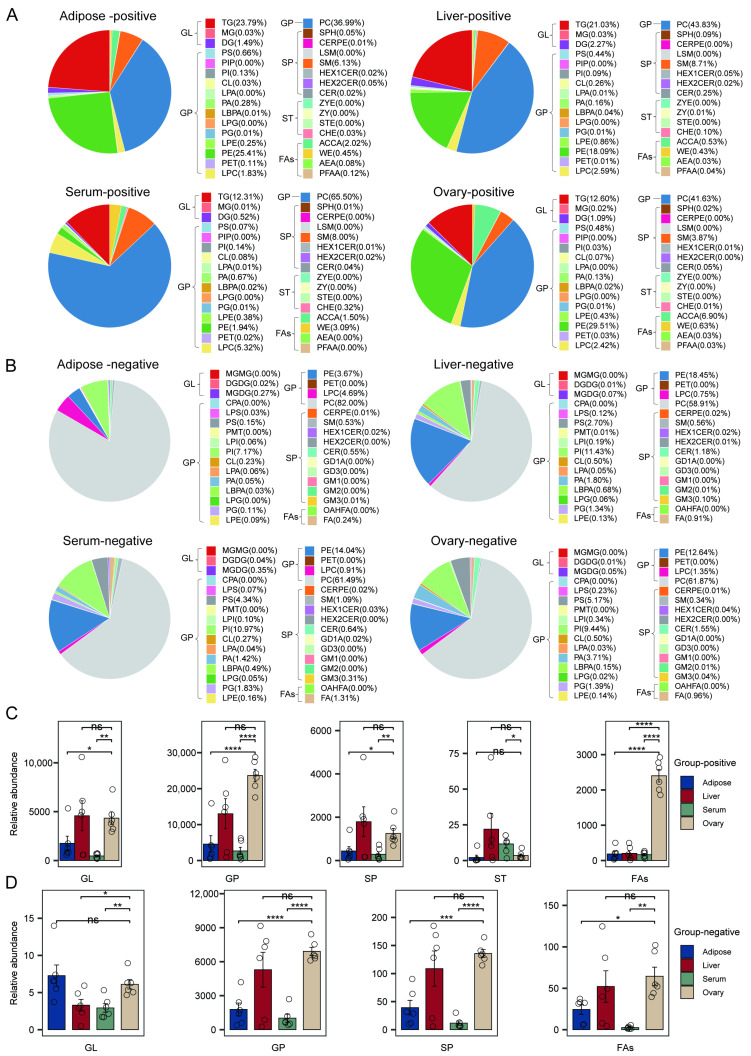
The proportions of lipid classes and subclasses of adipose tissue, liver, serum, and ovary in *H. bleekeri*. (**A**,**B**) Lipid profile of adipose tissue, liver, serum, and ovary. (**C**,**D**) Differences in lipid class composition among adipose tissue, liver, serum, and ovary in positive and negative ion modes. The results are reported as mean ± standard error (SEM). *n* = 6. * *p* < 0.05; ** *p* < 0.01; *** *p* < 0.001; **** *p* < 0.0001; ^ns^ *p* ≥ 0.05.

Correlation analysis demonstrated strong reproducibility among the QC samples in both the positive and negative ion modes. Furthermore, PCA score plots showed that the QC samples clustered tightly, confirming the stability and reliability of the analytical measurements (Figure S1). To further validate the robustness of the LC–MS/MS platform, representative total ion chromatograms (TICs) and base peak chromatograms (BPCs) were also examined. In the TIC profiles, the QC samples exhibited high overlap among injections, with consistent retention times, stable peak shapes, and no obvious signal drift or intensity fluctuation, which indicates the excellent stability and high reproducibility of the untargeted metabolomics workflow (Figure S2). Similarly, BPC analysis showed highly consistent peak patterns, retention behavior, and signal intensities across multiple QC runs, further confirming the robustness of the LC–MS/MS system and the reliability of the metabolomics data (Figure S3). Lipid composition analysis revealed that, under the positive ion mode, a total of five major classes of lipids were detected in adipose tissue, liver, serum, and ovary, among which GP exhibited the highest number of lipid classes. Further analysis showed that PC, TG, and PE accounted for the highest proportions among all lipid subclasses in adipose tissue, liver, and ovary; in serum samples, PC, TG, and sphingomyelin (SM) were the three most abundant lipid subclasses, accounting for 65.50%, 12.31%, and 8.00% of the total lipids, respectively (Figure 1A). Under the negative ion mode, a total of four major lipid classes were detected. In liver, serum, and ovary, PC, PE, and PI were the top three lipid subclasses; in adipose tissue samples, PC, PI, and lysophosphatidylcholine (LPC) were the most abundant, accounting for 82.00%, 7.17%, and 4.69% of the total lipids, respectively (Figure 1B).

Under the positive ion mode, the lipid levels in adipose tissue and serum were significantly lower than those in the ovary in the glycerolipids (GLs), GP, and sphingolipids (SP) classes (Figure 1C, *p* < 0.05), while no significant difference was observed between the liver and ovary (Figure 1C, *p* > 0.05). For the sterol lipids (STs) class, the liver exhibited the highest lipid content, and the serum lipid levels differed significantly from those in the ovary (Figure 1C, *p* < 0.05). Among the fatty acyls (FAs) class, ovarian lipids showed the highest content, which was significantly higher than that in the other three sample types (Figure 1C, *p* < 0.0001). Under the negative ion mode, the ovarian lipid levels were significantly higher than those in adipose tissue and serum in the GP, SP, and FAs classes (Figure 1D, *p* < 0.05), while no significant difference was observed between the liver and ovary (Figure 1C, *p* > 0.05). In the GL class, significant differences were found between the ovary and liver (*p* < 0.05), as well as between the ovary and serum (*p* < 0.01; Figure 1D). The abbreviations of all lipids identified in this study are provided in Table S2.

### 3.2. Lipid Molecules Associated with Ovarian Yolk Lipid Deposition

**Figure 2:**
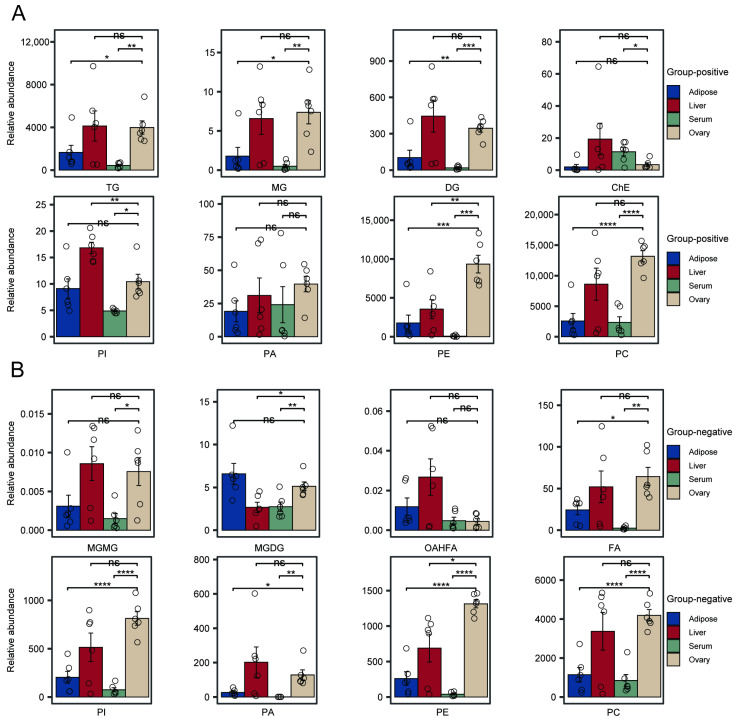
Distribution of yolk-deposition-related lipids in adipose tissue, liver, serum, and ovary. (**A**) Expression profiles of yolk-deposition-related lipids in different tissues under the positive ion mode. (**B**) Expression profiles of yolk-deposition-related lipids in different tissues under the negative ion mode. *n* = 6. * *p* < 0.05; ** *p* < 0.01; *** *p* < 0.001; **** *p* < 0.0001; ^ns^ *p* ≥ 0.05.

Under positive ion mode, the ovary showed no significant differences from the liver in the TG, MG, DG, ChE, PA, and PC subclasses (Figure 2A, *p* > 0.05), and it exhibited significantly higher lipid levels than adipose tissue and serum in the TG, MG, DG, PE, and PC subclasses (Figure 2A, *p* < 0.05), while no significant differences were observed in the PA subclass with any of the other tissues (Figure 2A, *p* > 0.05). Under negative ion mode, the ovary showed significant differences in multiple lipid subclasses compared to serum (MGMG, MGDG, FA, PI, PA, PE, and PC), liver (MGDG and PE), and adipose tissue (FA, PI, PA, PE, and PC), except for OAHFA (Figure 2B, *p* < 0.05).

### 3.3. Tissue-Specific Distribution of DHA- and ARA-Containing GPs and TGs

**Figure 3:**
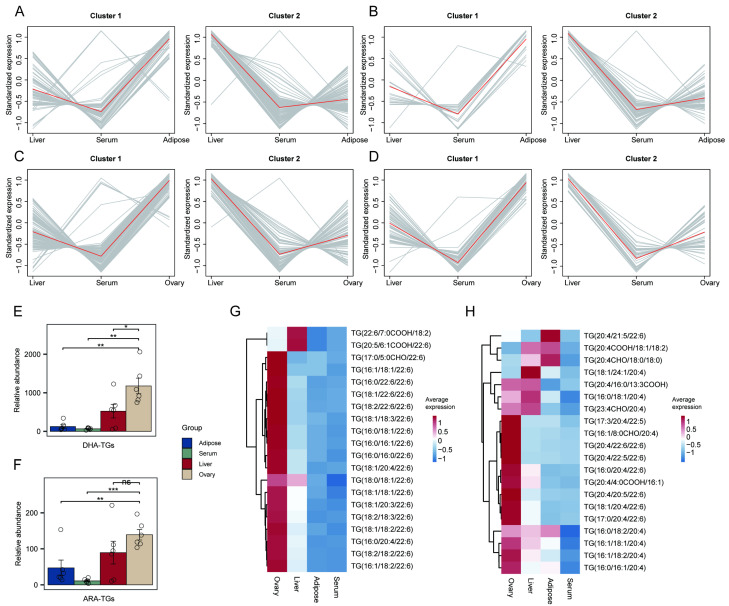
Enrichment patterns of DHA- and ARA-containing GPs and TGs across adipose tissue, liver, serum, and ovary. Mfuzz analysis of lipid expression patterns across liver, serum, and adipose tissue (**A**,**B**) or ovary (**C**,**D**). Red lines represent the mean expression trend of each cluster, and grey lines represent the expression profiles of individual lipids. Only lipids with an Mfuzz membership value greater than 0.6 were classified into eight clusters and displayed. (**E**,**F**) Tissue-specific enrichment of DHA- and ARA-containing TGs in adipose tissue, liver, serum, and ovary. * *p* < 0.05; ** *p* < 0.01; *** *p* < 0.001; ^ns^ *p* ≥ 0.05. (**G**,**H**) Heatmaps depicting the top 20 DHA- and ARA-containing TGs exhibiting the highest inter-tissue variation across adipose tissue, liver, serum, and ovary.

Mfuzz clustering analysis performed on DHA- and ARA-containing GPs showed that these GPs could be classified into two major clusters (cluster 1 and cluster 2) with opposite trends. A greater proportion of core DHA-containing GPs were assigned to cluster 2, which indicates their higher abundance in the liver (Figure 3A, Table S3); similarly, most core ARA-containing GPs were also enriched in cluster 2, which suggests their preferential accumulation in the liver (Figure 3B, Table S4). Further Mfuzz analysis focusing on the distribution of DHA- and ARA-containing GPs across the liver, serum, and ovary revealed that core DHA- and ARA-containing GPs were predominantly grouped into cluster 1 and were markedly enriched in the ovary (Figure 3C,D, Tables S5 and S6).

Among the four tissues examined, DHA- and ARA-containing TGs exhibited the highest abundance in the ovary, with enrichment levels significantly exceeding those in adipose tissue and serum (Figure 3E,F, ** *p* < 0.01). The heatmap of the top 20 lipids showed great inter-tissue variability across the four tissues (Figure 3G,H). With the exception of three DHA-containing TGs—TG (22:6/7:0COOH/18:2), TG (20:5/6:1COOH/22:6), and TG (17:0/5:0COOH/22:6)—all other DHA-containing TGs were markedly enriched in the ovary (Figure 3G). By contrast, among ARA-containing TGs, TG (20:4/21:5/22:6) and TG (20:4CHO/18:0/18:0) were preferentially enriched in adipose tissue, whereas TG (18:1/24:1/20:4) was more highly enriched in the liver (Figure 3H).

### 3.4. Differential Lipid Analysis Between Adipose Tissue and Ovary

**Figure 4:**
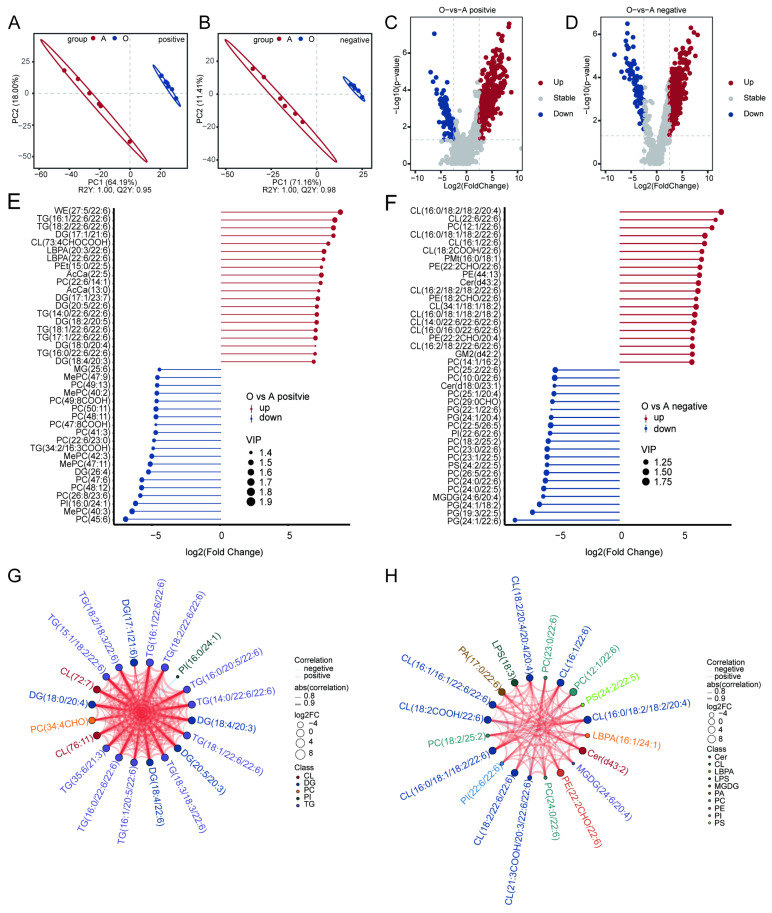
DELs between adipose tissue and ovary. (**A**,**B**) PCA analysis between adipose tissue and ovary under positive or negative ion mode separately. (**C**,**D**) Volcano map of DELs between adipose tissue and ovary under positive or negative ion mode separately. (**E**,**F**) Lollipop plot of DELs between adipose tissue and ovary under positive or negative ion mode separately. (**G**,**H**) Correlation of differential lipids between adipose tissue and ovary: positive ions in panel (**G**), negative ions in panel (**H**). O: ovary lipids, A: adipose lipids.

The liver is the main organ for lipid synthesis and secretion and delivers different lipids to adipose tissue and ovaries to perform distinct functions. An in-depth analysis of lipid class levels revealed marked differences between the groups for adipose and ovary in two ion models, with distinct lipid profiles identified between them using PCA (Figure 4A,B). A total of 653 differentially expressed lipids (DELs) were identified in positive ion mode, of which 547 were up-regulated and 106 were down-regulated, and 468 DELs were identified in negative ion mode, of which 374 were up-regulated and 94 were down-regulated, as shown in Figure 4C,D. The top 20 most up-regulated DELs in the ovary group consisted mainly of TGs and DGs in the positive ion model and CLs in the negative ion model (Figure 4E,F). In the positive ion model, correlation analysis of the top 20 DELs ranked by ascending *p*-value showed that, except for PI (16:0/24:1), which was significantly negatively correlated with the other differential lipids, the remaining lipids were positively correlated with each other (Figure 4G). However, in the negative ion model, most of the differential lipids showed negative correlations with each other (Figure 4H).

### 3.5. Functional Characterization of Lipids

**Figure 5:**
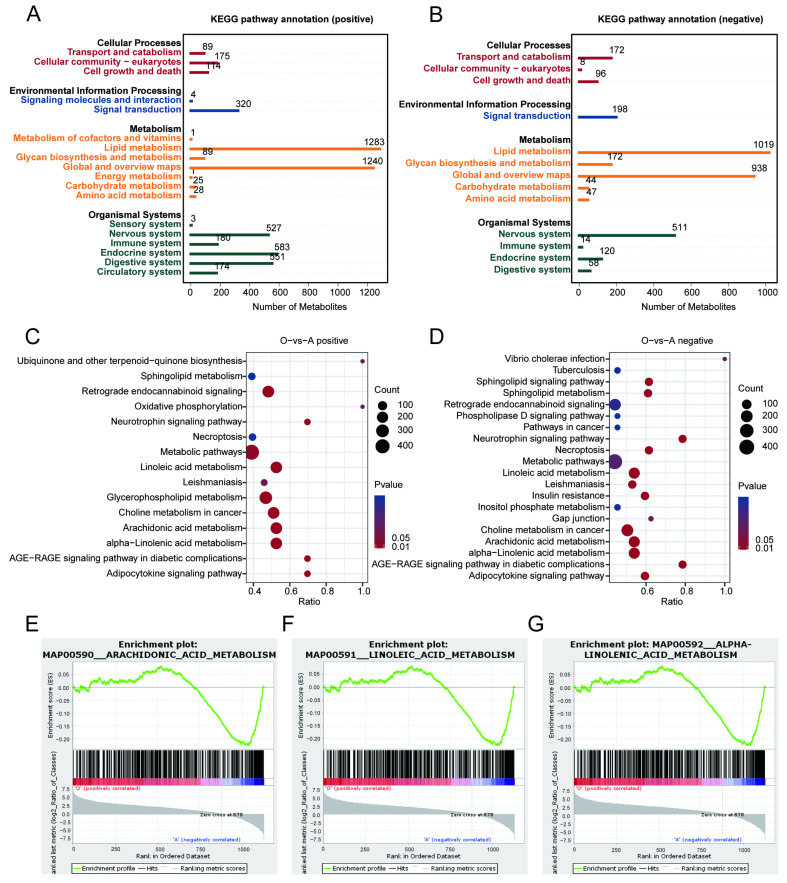
Lipid functional analysis. (**A**,**B**) KEGG pathway annotation of lipids under positive or negative ion mode separately. (**C**,**D**) KEGG enrichment analyses of lipids under positive or negative ion mode separately. (**E**–**G**) GSEA analysis of the arachidonic acid metabolism (MAP00590), linoleic acid metabolism (MAP00591), and alpha-linolenic acid metabolism (MAP00592) in lipidomics, respectively. O: ovary lipids, A: adipose lipids. Black vertical lines mark pathway hit lipid species. The color gradient bar illustrates lipid correlation: red = positive correlation, blue-purple = negative correlation. Green line shows enrichment score (ES).

KEGG classification revealed that lipids from adipose tissue, liver, serum, and ovary were primarily enriched in the metabolism category, with a particular emphasis on lipid metabolism and global and overview maps (Figure 5A,B). Compared to adipose tissue, the differential lipids were mainly enriched in metabolic pathways, glycerophospholipid metabolism, and glycerolipid metabolism (Figure 5C,D). Furthermore, GSEA based on the lipid abundance ranking showed that lipid species associated with arachidonic acid (MAP00590), linoleic acid (MAP00591), and alpha-linolenic acid (MAP00592) were predominantly enriched in adipose tissue (Figure 5E–G).

### 3.6. Tissue-Specific Expression Profiles of Lipid Metabolism Genes in the Liver, Adipose Tissue, and Ovary

**Figure 6:**
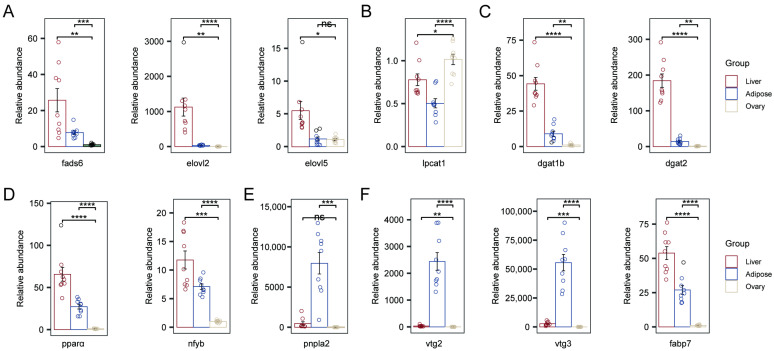
Tissue-specific expression profiles of key genes involved in lipid metabolism. Relative mRNA expression levels of genes related to (**A**) LC-PUFA biosynthesis (*fads6*, *elovl2*, *elovl5*), (**B**) phospholipid remodeling (*lpcat1*), (**C**) triglyceride synthesis (*dgat1b*, *dgat2*), (**D**) transcription factor (*pparα*, *nfyb*), (**E**) lipid mobilization (*pnpla2*) and (**F**) lipid transport (*vtg2*, *vtg3*, *fabp7*) across the liver, adipose tissue, and ovary. * *p* < 0.05; ** *p* < 0.01; *** *p* < 0.001; **** *p* < 0.0001; ^ns^ *p* ≥ 0.05.

To investigate the molecular basis underlying the high accumulation of DHA and ARA in the ovary, the expression profiles of key lipid metabolism-related genes were analyzed across tissues. The fatty acid desaturation and elongation genes fatty acid desaturase 6 (*fads6*), elongation of very-long-chain fatty acids protein 2 (*elovl2*), and elongation of very-long-chain fatty acids protein 5 (*elovl5*) were predominantly expressed in the liver (Figure 6A), whereas lysophosphatidylcholine acyltransferase 1 (*lpcat1*), a key gene involved in phospholipid remodeling and PUFA incorporation, was most highly expressed in the ovary (Figure 6B). In addition, the expression levels of the triglyceride synthesis genes diacylglycerol acyltransferase 1b (*dgat1b*) and diacylglycerol acyltransferase 2 (*dgat2*) were significantly higher in the liver and adipose tissue than in the ovary (Figure 6C, *p* < 0.01), accompanied by elevated expression of the lipogenic transcription factors peroxisome proliferator-activated receptor alpha (*pparα*) and nuclear transcription factor Y subunit beta (*nfyb*) in these tissues (Figure 6D, *p* < 0.001). Conversely, adipose tissue exhibited relatively high expression of patatin-like phospholipase domain-containing protein 2 (*pnpla2*) (Figure 6E, *p* < 0.001). Meanwhile, the transcript levels of vitellogenin 2/3 (*vtg2/3*) and fatty acid-binding protein 7 (*fabp7*) were markedly higher in the liver than in the ovary (Figure 6F, *p* < 0.0001).

## 4. Discussion

Lipids are essential organic compounds for maintaining various physiological activities and energy storage in fish and are extensively involved in metabolic processes, cell growth, and signal transduction [12,25,26]. In teleost fish, they are also prominent components of egg yolk and are, thus, a major source of nutrients for the embryo [27].

In this study, lipidomic analysis showed that GP was the predominant lipid class in the adipose tissue, liver, serum, and ovary of *H. bleekeri*, which is consistent with salmonid requirements for high phospholipid levels to promote fish growth and development [28]. Previous studies in teleost fish have demonstrated that PC is also the dominant polar lipid class in both the liver and ovary [29]; consistent with these findings, the present study showed that it was the most abundant lipid class in adipose tissue, liver, serum, and ovary among all detected lipids. Several studies have shown that PC and PE are the most abundant lipoprotein yolk phospholipids in fishes [30,31], and our results demonstrated a pronounced enrichment of both in the ovary relative to adipose tissue, liver, and serum, suggesting an ovary-specific accumulation of membrane phospholipids in *H. bleekeri*.

Dietary supplementation with DHA and ARA has been shown to influence the tissue fatty acid composition of aquatic animals. In the ovary, fertilized eggs, and larvae, the variation trends of ARA and DHA are generally consistent with those in the diet, indicating that tissue fatty acid profiles can reliably reflect dietary fatty acid composition, and their levels also exhibit a linear increase with increasing dietary ARA content [32]. In half-smooth tongue sole (*Cynoglossus semilaevis*), DHA has been shown to elevate estradiol (E2) levels in females, whereas ARA exhibits preferential accumulation in the gonads [33]. Furthermore, the latter has been shown to enhance fecundity in Japanese flounder (*Paralichthys olivaceus*) [34]. During vitellogenesis, lipids are accumulated in developing oocytes through coordinated processes involving hepatic synthesis, blood transport, and ovarian uptake. Under estrogen stimulation, the liver synthesizes vitellogenins and lipid-rich lipoproteins, which transport DHA-, ARA-, and other LC-PUFA-containing lipids to the ovary, where they are selectively retained and remodeled for yolk formation and oocyte development [12,35].

DHA in biological systems is predominantly incorporated into phospholipids, forming DHA-containing GPs such as PC and PE, which contribute primarily to membrane structure and function [36]. DHA-containing TGs represent the major storage form of DHA in tissues and yolk, serving as an important energy reserve and lipid source for early embryonic development [12]. Our results demonstrate a significant enrichment of DHA- and ARA-containing GPs and TGs in the ovary, highlighting the preferential allocation of these polyunsaturated fatty acids to ovarian membrane lipids, which supports active membrane biogenesis and yolk formation during oocyte development in *H. bleekeri*.

The relatively high hepatic expression of *fads2*, *elovl2*, and *elovl5* supports the liver as a major organ for endogenous LC-PUFA biosynthesis in *H. bleekeri*, consistent with previous reports in salmonids, *Siganus canaliculatus* and hybrid grouper (*Epinephelus fuscoguttatus* ♀ × *Epinephelus lanceolatus* ♂) [37,38,39]. The annotated *fads6* identified in *H. bleekeri* likely represents a teleost *fads2*-type Δ6 fatty acyl involved in LC-PUFA biosynthesis [40]. The present results showing high expression levels of key desaturase and elongase genes including *fads6*, *elovl2* and *elovl5* confirm that the liver is the primary site for LC-PUFA synthesis. Nevertheless, lipids containing DHA and ARA exhibited distinct tissue-specific redistribution patterns and were abundantly accumulated in the ovary [31,41]. Notably, in this study, the abundance of DHA- and ARA-containing triglycerides in the ovary was markedly higher than that in adipose tissue, which indicates that female broodstock preferentially allocate LC-PUFA-rich neutral lipids to ovarian deposition rather than long-term somatic energy storage during vitellogenesis.

*Dgat1* and *dgat2* catalyze the final and committed step of triacylglycerol biosynthesis and play essential roles in lipid deposition and energy storage [42]. In the present study, although the expression levels of the key triacylglycerol synthesis genes *dgat1b* and *dgat2* were relatively low in the ovary, it still showed the highest enrichment of DHA- and ARA-containing triacylglycerols, which suggests that lipid accumulation in the ovary relies more on the selective uptake and retention of LC-PUFAs rather than endogenous de novo lipid synthesis [29]. By contrast, the significantly up-regulated expression of *vtg2/3* and *fabp7* in the liver further supports its central role in yolk precursor synthesis and lipid transport during vitellogenesis [43]. Consistent with previous studies, this study further confirms that vitellogenins are mainly synthesized in hepatocytes and subsequently transported to developing oocytes. Notably, the relatively higher expression of *vtg2/3* detected in adipose tissue in the present study may additionally suggest potential extrahepatic involvement in vitellogenin-related lipid metabolism during ovarian development. Moreover, the markedly elevated ovarian expression of *lpcat1* suggests active phospholipid remodeling through the Lands cycle [44], which may facilitate the selective incorporation and retention of LC-PUFAs such as DHA and ARA into membrane phospholipids. This study’s high diversity of DHA- and ARA-containing glycerophospholipids in the ovary further demonstrates that ovarian lipid metabolism undergoes dynamic molecular remodeling rather than simple passive deposition.

As a signature phospholipid of the inner mitochondrial membrane, cardiolipin is closely associated with oxidative phosphorylation efficiency and mitochondrial biogenesis [45]. Its ovarian enrichment, together with the predominantly negative correlations observed among differential lipids under the negative ion mode, further suggests elevated mitochondrial membrane remodeling and oxidative metabolic activity associated with oocyte maturation and rapid cellular growth.

Overall, in this study, we found that LC-PUFA-rich lipids are selectively mobilized and preferentially allocated to reproductive tissues during vitellogenesis rather than serving solely as general energy reserves. Such targeted lipid redistribution may represent an important nutritional strategy for supporting oocyte maturation, yolk formation, and early embryonic development [41]. From the perspective of broodstock management and artificial propagation, this study highlights the importance of precisely regulating dietary LC-PUFAs, particularly DHA and ARA, in broodstock nutrition, which is of great practical significance for optimizing ovarian lipid deposition, improving egg and larval quality, enhancing artificial breeding efficiency, and overcoming bottlenecks in large-scale seed production [41]. However, the relative contributions of endogenous biosynthesis and dietary intake to LC-PUFA accumulation during vitellogenesis remain unclear. Future studies integrating lipidomics, enzyme activity assays, stable isotope tracing, and gene expression analyses of key LC-PUFA biosynthetic enzymes will help elucidate the underlying regulatory mechanisms governing LC-PUFA deposition and allocation. Furthermore, this study provides an important theoretical basis and nutritional reference for artificial stock enhancement, population recovery, and the conservation of wild resources of *H. bleekeri*.

## 5. Conclusions

This study revealed distinct tissue-specific lipid redistribution patterns in female *H. bleekeri* during vitellogenesis, indicating clear selective allocation of LC-PUFAs between somatic and reproductive tissues. DHA- and ARA-containing GPs were preferentially enriched in the ovary, which suggests the selective allocation of LC-PUFA-rich lipids to support membrane biogenesis, yolk formation, and oocyte development. The abundance of DHA- and ARA-containing TGs was also markedly higher in the ovary than in adipose tissue, which indicates that female broodstock preferentially allocate LC-PUFA-rich neutral lipids to ovarian deposition rather than long-term somatic energy storage during vitellogenesis. Mechanistically, the high hepatic expression of *fads6*, *elovl2*, *elovl5*, and *fabp7* highlights the central role of the liver in LC-PUFA biosynthesis and transport, whereas the up-regulation of ovarian *lpcat1* suggests active phospholipid remodeling and selective retention of fatty acids. Collectively, these findings provide a mechanistic basis for optimizing broodstock nutritional strategies, emphasizing the importance of dietary DHA and ARA regulation to improve reproductive performance and seed quality and offering implications for the artificial propagation and conservation of this endangered species.

## Data Availability

The original contributions presented in this study are included in the article/Supplementary Materials; further inquiries can be directed to the corresponding author.

## Supplementary Materials

The following supporting information can be downloaded at: https://www.mdpi.com/article/10.3390/biology15131059/s1, Figure S1: Data quality assessment. Figure S2: Total ion chromatograms (TICs) of QC samples and experimental samples under positive and negative ion modes. Figure S3: Base peak chromatograms (BPCs) of QC samples and test samples under positive and negative ion modes. Table S1: The sequence of primer pairs used in this study. Table S2: Lipid class abbreviations. Table S3: Mfuzz analysis of DHA-GPs expression patterns across liver, serum, and adipose tissue. Table S4: Mfuzz analysis of ARA-GPs expression patterns across liver, serum, and adipose tissue. Table S5: Mfuzz analysis of DHA-GPs expression patterns across liver, serum, and ovary. Table S6: Mfuzz analysis of ARA-GPs expression patterns across liver, serum, and ovary.
